# Differences between immunotherapy-induced and primary hypophysitis—a multicenter retrospective study

**DOI:** 10.1007/s11102-021-01182-z

**Published:** 2021-09-13

**Authors:** Felix Amereller, Timo Deutschbein, Mamta Joshi, Jochen Schopohl, Katharina Schilbach, Mario Detomas, Leo Duffy, Paul Carroll, Sophie Papa, Sylvère Störmann

**Affiliations:** 1 Medizinische Klinik und Poliklinik IV, LMU Klinikum, Ziemssenstr. 1, 80336 München, Germany; 2 Division of Endocrinology and Diabetes, Department of Internal Medicine I, University Hospital Würzburg, University of Würzburg, Würzburg, Germany; 3 Medicover Oldenburg MVZ, Oldenburg, Germany; 4 Department of Endocrinology, Guy’s & St. Thomas’ NHS Foundation Trust, London, UK; 5 School of Cancer and Pharmaceutical Sciences, King’s College London, London, UK

**Keywords:** Primary hypophysitis, Immunotherapy-induced hypophysitis, Checkpoint inhibitors, Immune**-**related adverse events, Pembrolizumab, Ipilimumab, Nivolumab

## Abstract

**Objective:**

Immune checkpoint inhibitors can cause various immune-related adverse events including secondary hypophysitis. We compared clinical characteristics of immunotherapy-induced hypophysitis (IIH) and primary hypophysitis (PH)

**Design:**

Retrospective multicenter cohort study including 56 patients with IIH and 60 patients with PH.

**Methods:**

All patients underwent extensive endocrine testing. Data on age, gender, symptoms, endocrine dysfunction, MRI, immunotherapeutic agents and autoimmune diseases were collected.

**Results:**

Median time of follow-up was 18 months in IIH and 69 months in PH. The median time from initiation of immunotherapy to IIH diagnosis was 3 months. IIH affected males more frequently than PH (p < 0.001) and led to more impaired pituitary axes in males (p < 0.001). The distribution of deficient adenohypophysial axes was comparable between both entities, however, central hypocortisolism was more frequent (p < 0.001) and diabetes insipidus considerably less frequent in IIH (p < 0.001). Symptoms were similar except that visual impairment occurred more rarely in IIH (p < 0.001). 20 % of IIH patients reported no symptoms at all. Regarding MRI, pituitary stalk thickening was less frequent in IIH (p = 0.009). Concomitant autoimmune diseases were more prevalent in PH patients before the diagnosis of hypophysitis (p = 0.003) and more frequent in IIH during follow-up (p = 0.002).

**Conclusions:**

Clinically, IIH and PH present with similar symptoms. Diabetes insipidus very rarely occurs in IIH. Central hypocortisolism, in contrast, is a typical feature of IIH. Preexisting autoimmunity seems not to be indicative of developing IIH.

## Introduction

Barely ten years ago, the immune-modulating monoclonal antibody ipilimumab was approved for the treatment of metastatic melanoma, heralding the introduction of immune checkpoint inhibition as solid tumor treatment [1]. This sustainably stimulated the field of immuno-oncology and marked a turning point in cancer therapy. To date, the FDA has approved several monoclonal antibodies for various solid tumors and development of new agents is ongoing. The basic mechanism of action relies on the inhibition of signals that physiologically downregulate immune responses, namely cytotoxic T lymphocyte antigen 4 (CTLA-4) and programmed cell death 1 (PD-1), effectively dis-inhibiting T cells [2, 3]. Side effects include derailed immune responses from an excessively activated immune system and these have been termed immune-related adverse events (irAE). Theoretically the whole spectrum of inflammatory symptoms and diseases may occur, including diarrhea, colitis, rash, arthritis, pneumonitis, hepatitis, myocarditis, and encephalitis. Immune checkpoint inhibitors (ICPI) may also lead to a multitude of endocrinopathies, such as thyroiditis, primary adrenal insufficiency, diabetes mellitus, and hypophysitis [4, 5]. Notably, hypophysitis is a frequent irAE [6–8], occurring in 5% of ICPI-treated patients according to a recent big data analysis [9].

The exact mechanisms that lead to irAE remain largely unclarified but several mechanisms are thought to be potential contributors. Organ-specific characteristics seem to play a role in the different patterns of drug toxicities [10, 11]. In cases of immunotherapy-induced hypophysitis (IIH), an autopsy study showed that anterior pituitary cells express CTLA-4, causing T-cell infiltration, IgG-dependent complement fixation, and phagocytosis under CTLA-4 blockade [12]. Inflammation was more pronounced in patients expressing higher levels of CTLA-4 antigen in the pituitary [12]. One study found that CTLA-4 blockade in a murine model led to lymphocytic infiltration in the pituitary [13]. Furthermore, in a cohort of 20 patients receiving CTLA-4-targeted ipilimumab, seven patients developed pituitary antibodies and a clinical diagnosis of hypophysitis [13].

In contrast, primary hypophysitis (PH) may involve an array of different pathological lesions, of which lymphocytic infiltration is the most common [14]. However, given the similarities between both forms of hypophysitis and the multitude of unanswered questions surrounding their exact mechanisms, whether they share a common pathway remains unclear. So far, clinical descriptions of patients with hypophysitis have focused on either IIH or PH. Comparisons between studies are difficult due to inherent methodological differences. To address this knowledge gap, we directly compared patients with acquired hypophysitis secondary to ICPI therapy with those with the primary autoimmune form, using the same methods for each cohort.

## Methods and patients

This cohort study was conducted at three tertiary referral centers, two in Germany and one in the UK. The participating centers’ databases were searched for patients diagnosed with IIH between 2010 and 2020. Eligible patients were those who had received at least one dose of ICPI and had presented in the endocrinology department where they received a complete endocrine assessment (see below). Patients with a follow-up time of less than a month were excluded. Patients’ complete medical records were analyzed, and a clinical expert (JS, TD, PC) thoroughly reviewed each case. Local expert radiologists analyzed the MRIs. A diagnosis of IIH was established when pituitary dysfunction and typical MRI signs of hypophysitis occurred in patients with ongoing or previous cancer immunotherapy. Data were collected retrospectively using the same data sheet in each center and for both cohorts. The cohort of IIH patients was compared to a similar sized cohort of PH patients in one of the participating centers (Munich).

### Endocrine assessment

All patients underwent extensive endocrine laboratory testing on their first presentation at participating centers. Testing included measuring serum morning cortisol, morning ACTH, cortisol after stimulation with synthetic ACTH, FSH, LH, estrogen, testosterone, TSH, free T3 and free T4, IGF-I, IGFBP-3, fasting GH, and prolactin. In cases where GH deficiency was suspected, a stimulation test was performed (insulin tolerance test or GHRH + arginine test). Local laboratory cut-offs were used for the evaluation of hormone levels. All cut-off values were based on manufacturer’s data and validated for the locally used assays.

Patients were defined as having central hypothyroidism when they had undetectable TSH levels and low free thyroxine levels. Hypogonadotropic hypogonadism was defined as low LH and FSH levels in oligo- or amenorrhoeic women or men with low testosterone levels. ACTH-stimulated cortisol levels were used for the evaluation of corticotroph function. In edge cases, additional insulin tolerance tests were performed. Low random GH and IGF-I levels were considered to be related to GH deficiency and the diagnosis established if three other pituitary hormone deficiencies were present. In all other cases, provocative testing was performed. Assessments of serum and urine osmolality and sodium levels after overnight fasting were used to screen for central diabetes insipidus (DI). In patients with polydipsia and polyuria, DI was determined if osmolality was below 400 mosm/kg in urine and elevated in serum. Patients with clinical signs of DI but inconclusive laboratory assessments underwent a water deprivation test. Patients were screened for thyroid autoantibodies at diagnosis and during follow-up if hyper- or hypothyroidism developed. The number of impaired axes was counted for each patient, considering central diabetes insipidus (DI) as well as hypoprolactinemia as an impaired axis each.

### Collected data

All analyzed items are listed in Table 1. NA (“not available”) was entered when the information was either not assessed or unclear.

**Table 1:** Collected data

General information	Age at diagnosis, gender, time of follow-up
Symptoms at diagnosis	Headache (no/yes), fatigue (no/yes), dizziness (no/yes), nausea (no/yes), emesis (no/yes), adrenal crisis (no/yes), hyponatremia (no/yes), hypernatremia (no/yes), polyuria (no/yes), polydipsia (no/yes), amenorrhoea (no/yes), loss of libido (no/yes), erectile dysfunction (no/yes), hypotension (no/yes), hypoglycaemia (no/yes), neurological disorder (no/yes), visual impairment (no/yes), fever (no/yes)
Endocrine dysfunction	Secondary hypothyroidism (no/yes), secondary adrenal insufficiency (no/yes), diabetes insipidus (no/yes), secondary hypogonadism (no/yes), GH deficiency (no/yes), hyperprolactinemia (no/yes), hypoprolactinemia (no/yes)
Magnetic resonance imaging (MRI) findings	MRI prior to diagnosis and during follow-up, if available, were analysed as well. The following items were assessed: pituitary size (normal/enlarged/reduced), size of pituitary stalk (normal/thickened), gadolinium contrast enhancement (weak/strong), empty sella (no/yes), intrasellar space occupying lesion (no/yes), cystic lesion (no/yes), central necrosis (no/yes), optic chiasm affected, other abnormalities of the pituitary or comments (free text)
Cancer immunotherapy	Immunotherapy agent, time from initiation of immunotherapy treatment to diagnosis of hypophysitis, number of doses administered prior to diagnosis of hypophysitis, indication for immunotherapy
Concomitant autoimmune diseases	Concomitant autoimmune disease prior to diagnosis of hypophysitis (no/yes) or during follow up (no/yes), thyroid ultrasound performed (no/yes), thyroid antibodies measured (no/yes), thyroiditis during follow-up (no/yes)

### Patients

Fifty-six patients with IIH were included in this study and data was retrospectively compared to that of a cohort of 60 patients with PH, which was previously reported elsewhere [15]. The median time of follow-up was 18 ± 15 months in IIH patients and 69 ± 75 in PH patients.

### Statistical analysis

Statistical analyses were performed using IBM SPSS Statistics version 25 (IBM Corporation, Armonk, NY, U.S.A.). Group differences were calculated using a Mann-Whitney U test and either a chi-square test or Fisher’s exact test. P values of ≤ 0.05 were considered significant. Regarding the comparison of symptoms, items were excluded from the analysis when data was missing in ≥ 20 % of patients in one or both groups.

### Ethical and data safety considerations

Data were collected at the Guy’s and St Thomas’ NSH Foundation Trust as part of a locally approved audit. In Munich and Würzburg, informed consent approving the use of clinical data for research purposes was obtained from all patients during a visit to the outpatient clinic. The data were compiled anonymously in the study file. The ethical standards defined by the respective institutional research boards, the ICH GPC guidelines, and the 1964 Declaration of Helsinki and all subsequent amendments were followed.

## Results

### Cancer immunotherapy

Immune checkpoint inhibitors found to cause hypophysitis were ipilimumab (n = 24), ipilimumab/nivolumab (n = 18), pembrolizumab (n = 10), nivolumab (n = 3), and ipilimumab/pembrolizumab (n = 1). The median number of doses administered before the diagnosis of IIH was four (range 2–14). Median time to toxicity was six months in patients receiving PD-1 blockade (n = 13), three months under CTLA-4 blockade (n = 24), and three months under CTLA-4/PD-1 blockade (n = 19).

### Demographic characteristics

Age and gender distribution differed between IIH and PH patients (cf. Table 2). Mean age at diagnosis was higher in IIH patients (60 years) than in PH patients (45 years) (p < 0.001). While IIH was more frequent in males (64%) and the median age of onset did not differ between genders, women were more frequently affected by PH (73% women, p < 0.001) and tended to be affected at a younger age than men.

**Table 2:** General characteristics of both cohorts

Characteristic	IIH (n = 56)	PH (n = 60)	*p*-value
Male sex - no./total no. (%)	36/56 (64)	16/60 (27)	**< 0.001**
Age at diagnosis – yr (range)	60 ± 14 (22–87)	45 ± 16 (15–83)	**< 0.001**
Male	60.5	49.5.	n.a.
Female	61.5	40.0	n.a.
Time of follow-up - months	18 ± 15	69 ± 75	n.a.

Bold values indicate *p* < 0.05

### Symptoms

Table 3 displays the frequency of symptoms in both cohorts. Overall, the clinical presentation was similar between the groups, but visual impairment (6% vs. 17%) and polyuria with polydipsia (4% vs. 38%) occurred more rarely in IIH than in PH (both p < 0.001). Notably, 20% of IIH patients reported no symptoms at all.

**Table 3:** Comparison of symptoms at diagnosis between IIH and PH

Symptoms	IIH	PH	*p*-value
Fatigue	66%	52%	n.s.
Headache	23%	38%	n.s.
Nausea	23%	17%	n.s.
Emesis	11%	12%	n.s.
Dizziness	22%	10%	n.s.
Adrenal crisis	13%	12%	n.s.
Visual impairment	6%	17%	**< 0.001**
Polyuria with polydypsia	4%	38%	**< 0.001**

Bold values indicate *p* < 0.05

### Endocrine dysfunction

The severity of pituitary dysfunction, defined by the number of impaired hormone axes, did not differ between the cohorts. Moreover, the order of frequency of adenohypophysial hormone deficiencies was the same in both groups: corticotropic (89% in IIH; 67% in PH; p < 0.001) > thyrotropic > gonadotropic > somatotropic (cf. Table 4). DI was rarely found in IIH, affecting only 4% of patients undergoing ipilimumab treatment but 38% of PH patients (p < 0.001).

**Table 4:** Comparison of endocrine dysfunction between IIH and PH

Impaired pituitary axes	IIH	PH	*p*-value
Corticotropic	89%	67%	**< 0.001**
Thyrotropic	55%	57%	n.s.
Gonadotropic	51%	52%	n.s.
Somatotropic	17%	20%	n.s.
Hypoprolactinemia	15%	3%	**0.033**
Hyperprolactinemia	13%	25%	n.s.
Diabetes insipidus	4%	38%	**< 0.001**
Median number of impaired pituitary axes (n)	2	2	n.s.

Bold values indicate *p* < 0.05

Hypoprolactinemia was more frequent in IIH than in PH (15% vs. 3 %, p = 0.033). Patients presenting with hypoprolactinemia had a median of three further hormone deficiencies, while the median number of hormone deficiencies in patients with normal prolactin levels was 2 (p = 0.043). Male patients had more impaired hormone axes. As displayed in Table 5, secondary hypothyroidism (p = 0.022) and secondary hypogonadism (p < 0.001) were more frequent in males than in females.

**Table 5:** Gender-specific endocrine dysfunction in IIH

Impaired pituitary axis	Females	Males	*p*-value
Corticotropic	90%	88%	n.s.
Thyrotropic	35%	66%	**0.022**
Gonadotropic	21%	66%	**< 0.001**
Somatotropic	11%	21%	n.s.
Hypoprolactinemia	5%	19%	n.s.
Hyperprolactinemia	5%	17%	n.s.
Diabetes insipidus	5%	3%	n.s.
Median number of impaired pituitary axes (n)	1.0	3.0	**< 0.001**

Bold values indicate *p* < 0.05

### MRI findings

For patients diagnosed with IIH, MRI scans of the sellar region were available in 84% of cases and an enlarged pituitary gland was found in 36% of cases. A thickened pituitary stalk (p = 0.009) and space-occupying intrasellar lesions or pituitary enlargement (p = 0.020) were less common in IIH than PH (cf. Table 6).

**Table 6:** Comparison of MRI findings between IIH and PH

MRI findings	IIH	PH	*p*-value
Enlarged pituitary size	36%	37%	n.s.
Reduced pituitary size	5%	10%	n.s.
Stalk thickening	27%	56%	**0.009**
Intrasellar lesion	27%	46%	**0.020**

Bold values indicate *p* < 0.05

In one patient, IIH was diagnosed solely based on MRI findings. The patient had experienced no symptoms and his pituitary function remained normal throughout follow-up. However, MRI performed for staging after four cycles of ipilimumab displayed an enlarged pituitary with a symmetrical and homogenously contrast-enhanced mass with a tent-shaped appearance in the coronal plane. Interestingly, the pituitary appeared completely unremarkable in the following MRI three months later: Pituitary size had returned to normal and no lesion was detectable. In this case, cerebral metastases never appeared, either initially or during the six years of follow-up. It is thus unlikely that the finding was a pituitary metastasis. Given the time-course, typical radiological appearance, and spontaneous remission, hypophysitis remains the most likely diagnosis.

### Concomitant autoimmune diseases

Autoimmune diseases before diagnosis of hypophysitis were less prevalent in IIH patients (13%) than PH patients (38%) (p = 0.003). In contrast, during follow-up, autoimmune diseases occurred more often in IIH patients (38% vs. 13%, p = 0.002). Autoimmune thyroiditis was the most prevalent entity affecting 18% of IIH patients and 33% of PH patients.

## Discussion

In this study, a cohort of 56 patients with IIH was retrospectively compared to a cohort of 60 patients with PH. The objective was to determine clinical differences between both entities. This is, to our knowledge, the first study directly comparing patients with each form of hypophysitis from cohorts of patients treated at the same investigational sites. Besides our analysis, only one study has compared the clinical aspects of PH and IIH. In 2016, Caturegli et al. performed a review of published cases, describing a heterogeneous cohort that was potentially subjected to selection and publication bias [12]. In contrast, we used the same methods of data collection for both cohorts and our resulting data were therefore homogenous and comparable.

Demographic data of our patients are in line with previously published cohorts [12, 16–18]. Age at diagnosis was higher in the IIH group, consistent with the increased cancer incidence at higher age that necessitates immunotherapy. While females are generally more prone to autoimmune diseases than males—and this hold true for PH patients—IIH more frequently affected males in this and previous studies [12, 18]. The reason for this gender difference is not yet understood. In general, immune response to auto-antigens seems less pronounced in males, accounting for their lower prevalence of autoimmune diseases [19]. If this is at least partly due to there being stronger activity of immune checkpoints in males, immune checkpoint inhibition might unleash an even more excessive immune response in males, thus causing more secondary autoimmune disorders.

The clinical presentation of both patient groups was comparable, featuring fatigue, headaches, and nausea. Notably, those symptoms are also frequent in cancer patients, and therefore we may have an overlap of symptoms from the underlying cancer and its treatment in IIH patients. Visual impairment occurred almost exclusively in patients with PH. This is clinically relevant as it is one of the most severe symptoms of hypophysitis and often requires surgical intervention. DI with polyuria and polydipsia was barely found in IIH patients. Congruently, stalk thickening was observed much less in IIH than in PH, hinting at a higher frequency of inflammatory dysfunction of the neurohypophysis in PH patients. IIH on the other hand can be clinically categorized as an inflammation of the adenohypophysis, which is reflected by the deficient axes depicted in our data.

The number of deficient axes and the order of hormone deficiencies of the anterior pituitary lobe (corticotropic > thyrotropic > gonadotropic > somatotropic) did not differ between groups. IIH and PH patients both frequently harbor secondary adrenal insufficiency, but in direct comparison, secondary adrenal insufficiency was more frequent in IIH than PH. As the vast majority of IIH patients had a deficient corticotropic axis, it can be considered a typical feature of IIH. In line with this, a study in Japan investigated 174 patients with non-small cell lung cancer treated with immunotherapy, and found that 16 patients developed pituitary irAE, all of which had ACTH deficiency [20]. Visual impairments due to pituitary swelling were more common in primary hypophysitis, and this supports the notion that hypocortisolism in IIH is not primarily caused by compression of corticotroph tissue. ACTH deficiency in cancer immunotherapy has also been linked to anti-corticotroph antibodies and specific HLA types [21].

Despite the prevalence of prolactin deficiency being low in both groups, it occurred more frequently in IIH and seemed associated with a more severe dysfunction of the adenohypophysis. One in five patients was GH deficient. So far, data on the somatotropic axis in patients with IIH are scarce [6]. However, the clinical implication of this is unclear, especially since most clinicians are reluctant to initiate rhGH treatment in the context of active malignancy.

Patients with IIH suffered less frequently from other autoimmune diseases than PH patients (13% vs. 38%, p = 0.003) [14]. To our knowledge, we present first-time data on the prevalence of autoimmune disease in IIH patients before ICPI treatment initiation. The prevalence of autoimmune diseases in the general population is estimated to be around 5–10% [22, 23], a number that is only slightly lower than that in our IIH cohort. Thus, preexisting autoimmunity seems not to indicate the development of hypophysitis following ICPI therapy.

This study has some limitations. Foremost, its retrospective nature means its ability to answer the pressing questions arising from the ongoing clinical experience with ICPI therapy was limited. The patients were scattered across three expert centers in two countries, which may have introduced geographical and ethnic confounding as well as biases of partial data or different clinical management. The latter is exemplified by the fact that MRI scans in some patients with IIH were omitted due to inconspicuous staging CAT scans and typical clinical course. In these cases, we cannot rule out pituitary metastasis as differential diagnosis with certainty but given the reversibility of symptoms and the lack of primary tumor mass as well as metastases in other regions, it seems highly unlikely. Thus, we feel confident that including these patients in our cohort of IIH does not introduce a bias. Furthermore, including patients from different centers with clear signs of hypophysitis could eliminate the systematic selection bias often found in monocentric analyses. Diagnoses were established according to local criteria, which may have led to a better generalizability in the clinical setting. Despite assembling the data from patients at three sites and thus achieving a large amount of patient data, we could not reasonably perform certain subgroup analyses such as comparison of clinical features of immunotherapy-induced hypophysitis depending on the compound used. However, overall, the number of patients included in the study allowed for sound descriptive and comparative statistics.

## Conclusions

Clinically, IIH and PH present with similar symptoms but DI is rare in IIH. Secondary hypocortisolism seems to occur in almost all patients with IIH. The distribution of deficient axes of the adenohypophysis is comparable between both entities and preexisting autoimmunity seems not to be indicative of developing IIH. Our findings confirm that the different causes of disease actually lead to different clinical features in these otherwise very similar autoimmune inflammations of the pituitary.

## Data Availability

The datasets analysed during the current study are available from the corresponding author after reconcilement with each site on reasonable request.

## Declarations

### Reporting guidelines

This study was reported in accordance with the STROBE (Strengthening the Reporting of Observational Studies in Epidemiology) recommendations.
